# Impairment of Spike-Timing-Dependent Plasticity at Schaffer Collateral-CA1 Synapses in Adult APP/PS1 Mice Depends on Proximity of Aβ Plaques

**DOI:** 10.3390/ijms22031378

**Published:** 2021-01-30

**Authors:** Machhindra Garad, Elke Edelmann, Volkmar Leßmann

**Affiliations:** 1 Institute of Physiology, Medical Faculty, Otto-von-Guericke University Magdeburg, 39120 Magdeburg, Germany; machhindra.garad@med.ovgu.de (M.G.); e.edelmann@physiologie.uni-kiel.de (E.E.); 2 Center for Behavioral Brain Sciences (CBBS), 39120 Magdeburg, Germany

**Keywords:** adult animals, Alzheimer, timing-dependent LTP, Schaffer collateral-CA1 synapses, amyloid beta plaques, fingolimod, FTY720

## Abstract

Alzheimer’s disease (AD) is a multifaceted neurodegenerative disorder characterized by progressive and irreversible cognitive decline, with no disease-modifying therapy until today. Spike timing-dependent plasticity (STDP) is a Hebbian form of synaptic plasticity, and a strong candidate to underlie learning and memory at the single neuron level. Although several studies reported impaired long-term potentiation (LTP) in the hippocampus in AD mouse models, the impact of amyloid-β (Aβ) pathology on STDP in the hippocampus is not known. Using whole cell patch clamp recordings in CA1 pyramidal neurons of acute transversal hippocampal slices, we investigated timing-dependent (t-) LTP induced by STDP paradigms at Schaffer collateral (SC)-CA1 synapses in slices of 6-month-old adult APP/PS1 AD model mice. Our results show that t-LTP can be induced even in fully developed adult mice with different and even low repeat STDP paradigms. Further, adult APP/PS1 mice displayed intact t-LTP induced by 1 presynaptic EPSP paired with 4 postsynaptic APs (6× 1:4) or 1 presynaptic EPSP paired with 1 postsynaptic AP (100× 1:1) STDP paradigms when the position of Aβ plaques relative to recorded CA1 neurons in the slice were not considered. However, when Aβ plaques were live stained with the fluorescent dye methoxy-X04, we observed that in CA1 neurons with their somata <200 µm away from the border of the nearest Aβ plaque, t-LTP induced by 6× 1:4 stimulation was significantly impaired, while t-LTP was unaltered in CA1 neurons >200 µm away from plaques. Treatment of APP/PS1 mice with the anti-inflammatory drug fingolimod that we previously showed to alleviate synaptic deficits in this AD mouse model did not rescue the impaired t-LTP. Our data reveal that overexpression of APP and PS1 mutations in AD model mice disrupts t-LTP in an Aβ plaque distance-dependent manner, but cannot be improved by fingolimod (FTY720) that has been shown to rescue conventional LTP in CA1 of APP/PS1 mice.

## 1. Introduction

Alzheimer’s disease (AD) is an age-related, multifaceted neurodegenerative disorder characterized by a progressive and irreversible cognitive decline. It is the most common cause of dementia and currently there is no disease-modifying therapy [1]. The main pathological hallmarks in AD are the presence of extracellular amyloid beta (Aβ) plaques, consisting of Aβ protein oligomers aggregates (Aβo, residues 1-40/42) and the accumulation of neurofibrillary tangles within neurons, composed of abnormally hyper-phosphorylated tau protein [2,3,4,5,6]. The Aβ plaques induce microglial activation, cytokine release, reactive astrocytosis, and subsequently an induction of chronic neuroinflammation, leading to increased levels of pro-inflammatory cytokines, neurotoxic tryptophan metabolites (kynurenines), and anti-inflammatory cytokines (compare review [7]). Importantly, neuroinflammatory signals mediated by microglial cells and concomitant astrogliosis are major hallmarks of AD [8,9]. Consequently, anti-inflammatory treatment of AD mice has been shown to be beneficial to counteract AD related synaptic and memory deficits (see [10]).

Degeneration of the medial temporal lobe, a critical brain region involved in memory formation, is a well-known indicator for AD [11,12,13]. The medial temporal lobe system comprises the hippocampal region and the adjacent entorhinal, perirhinal, and parahippocampal cortices (reviewed in [14]). Particularly, the memory impairments in the hippocampus are obvious in the early stage of AD [15,16]. The cornu ammonis (CA) 1 hippocampal region is crucial for spatial orientation, learning and different memory functions. It has been described that CA1 is one of the most affected regions in early stages of AD [17,18].

During development, critical periods of synaptic plasticity contribute to the formation and refinement of neural connections that are crucial for establishing synaptic circuits responsible for information storage in the adult brain. Long-term potentiation (LTP) is a dynamic enhancement of synaptic efficacy in the hippocampus and other brain areas, which is generally considered as a cellular correlate of learning and memory [19,20]. Spike timing-dependent plasticity (STDP) is a Hebbian form of synaptic plasticity and a physiological paradigm to induce LTP. It is induced by precisely timed (nearly) coincident firing of action potentials (APs) in pre- and post-synaptic neurons. While forward pairing (i.e., presynaptic neuron fires before postsynaptic cell) induces timing-dependent (t-)LTP, the backward sequence (postsynaptic neuron fires an AP before the presynaptic cell) induces t-LTD (reviewed in [21]). These bidirectional changes in synaptic efficacy have also been reported in hippocampal CA1 neurons where they are induced in response to low frequency repeated stimulation according to these STDP rules [22,23,24,25,26].

Although several studies have described that AD pathology in its early stage disrupts high frequency tetanus induced conventional LTP in the hippocampus (reviewed in [10,27,28,29,30]), its impact on STDP in the hippocampus remained elusive. Therefore, we assessed whether t-LTP, evoked by pairing of pre- and postsynaptic APs, at excitatory Schaffer collateral (SC)-CA1 synapses is changed in 6-month-old Alzheimer model mice carrying the KM670/671NL amyloid precursor protein (APP) and the L166P presenilin 1 mutations that are characteristic for familial Alzheimer’s disease (APP⁄PS1 mice; [31]). Since t-LTP induction protocols can vary with respect to their efficiency to induce synaptic changes and regarding their sensitivity to pathophysiological changes, we compared milder and therefore physiologically relevant low repeat (6×) STDP paradigms with stronger high repeat STDP paradigms (100×/35×), bearing in mind that robust stimulation induced t-LTP might be more sensitive to amyloidosis in adult APP/PS1 mice. However, our patch clamp STDP recordings demonstrated that t-LTP at SC-CA1 synapses can be induced with similar efficiency in hippocampal slices from 6-month-old APP/PS1 mice and wild-type littermates, when using 6× 1:4 or 100× 1:1 stimulation. Consequently, we argued that proximity of Aβ plaques to the recorded neuron might be decisive to observe a t-LTP deficit in APP/PS1 mice. In fact, we found that the 6× 1:4 t-LTP magnitude was significantly reduced if the recorded CA1 pyramidal neuron was located in the vicinity of Aβ plaques in APP/PS1 mice. Since microglia mediated neuroinflammation is a hallmark in AD mouse models, we tested whether chronic treatment with the anti-inflammatory drug Fingolimod (FTY720) that can alleviate other synaptic deficits in AD mice [10] could restore impaired t-LTP in these mice—but we did not observe such a rescue of t-LTP. Finally, we observed that the impaired t-LTP in CA1 neurons close to Aβ plaques was not due to changes in neuronal excitability or basal synaptic transmission in the recorded CA1 neurons.

## 2. Results

Although synaptic dysfunctions such as alterations in conventional long-term potentiation (LTP) and long-term depression (LTD) of hippocampal synaptic transmission have been described as an early event in Alzheimer disease (AD) mouse models (reviewed in [27]), it has not been previously reported whether AD alters spike timing-dependent plasticity (STDP) in the hippocampal formation. We used whole-cell patch clamp recordings in single postsynaptic cornu ammonis (CA) 1 pyramidal neurons from 6-month-old male C57Bl6/J wild-type (WT), APP/PS1 mice [31] and their WT littermates to study STDP at fully developed Schaffer collateral (SC)-CA1 synapses in 6-month-old adult animals. We tested different STDP paradigms to induce synaptic plasticity by repeatedly pairing a single presynaptic stimulation with either 1 or 4 postsynaptic action potentials (APs) with 10 ms interval (spike timings, Δt).

### 2.1. Low Repeat STDP Paradigms Induced Timing-Dependent (t-) LTP in Adult WT Mice

**Figure 1:**
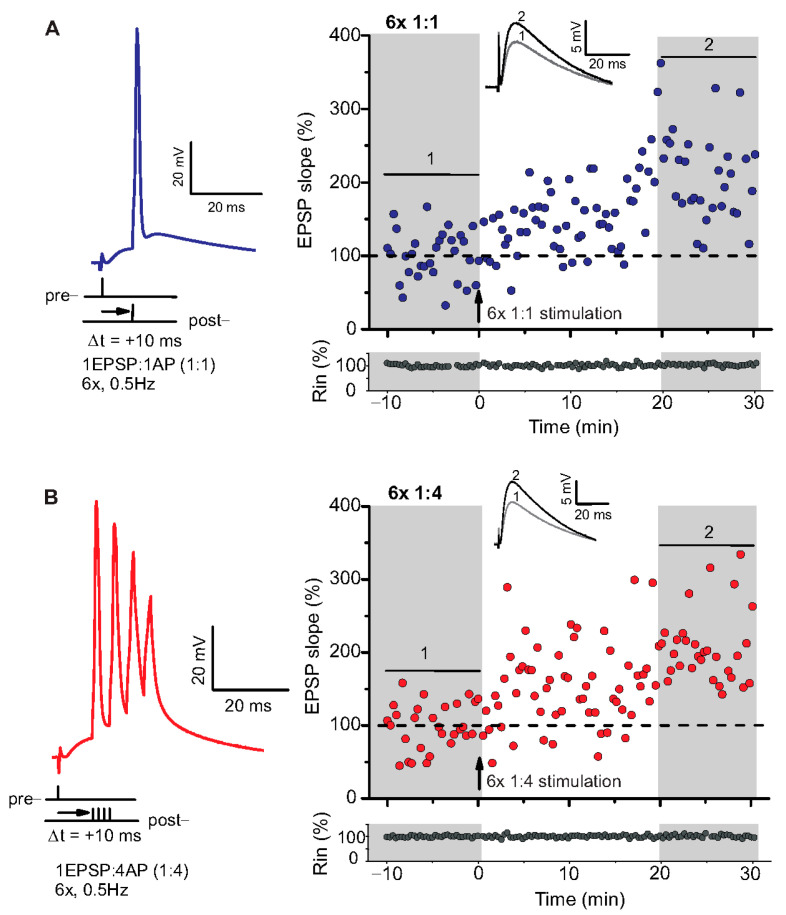
Spike timing-dependent LTP (t-LTP) can be induced with distinct low repeat spike timing-dependent plasticity (STDP) paradigms in 6-month-old adult mice. Whole cell patch clamp recordings (in current clamp) of timing-dependent LTP at SC-CA1 synapses of hippocampal slices from wild type mice. (**A**) One presynaptic EPSP paired with 1 postsynaptic action potential (AP, 6× 1:1, left inset) STDP paradigm used to induce canonical t-LTP. Typical recording from an individual cell for 6× 1:1 stimulation (indicated by arrow) induced t-LTP at SC-CA1 synapses. (**B**) One presynaptic EPSP paired with 4 postsynaptic APs (6× 1:4 protocol, left inset) STDP paradigm used to induce burst t-LTP. Typical recording from an individual cell for 6× 1:4 stimulation (indicated by arrow) induced t-LTP at the hippocampal SC-CA1 synapses. Insets: average EPSP before (1) and after t-LTP induction (2). Scale bars are shown in the respective insets.

**Figure 2:**
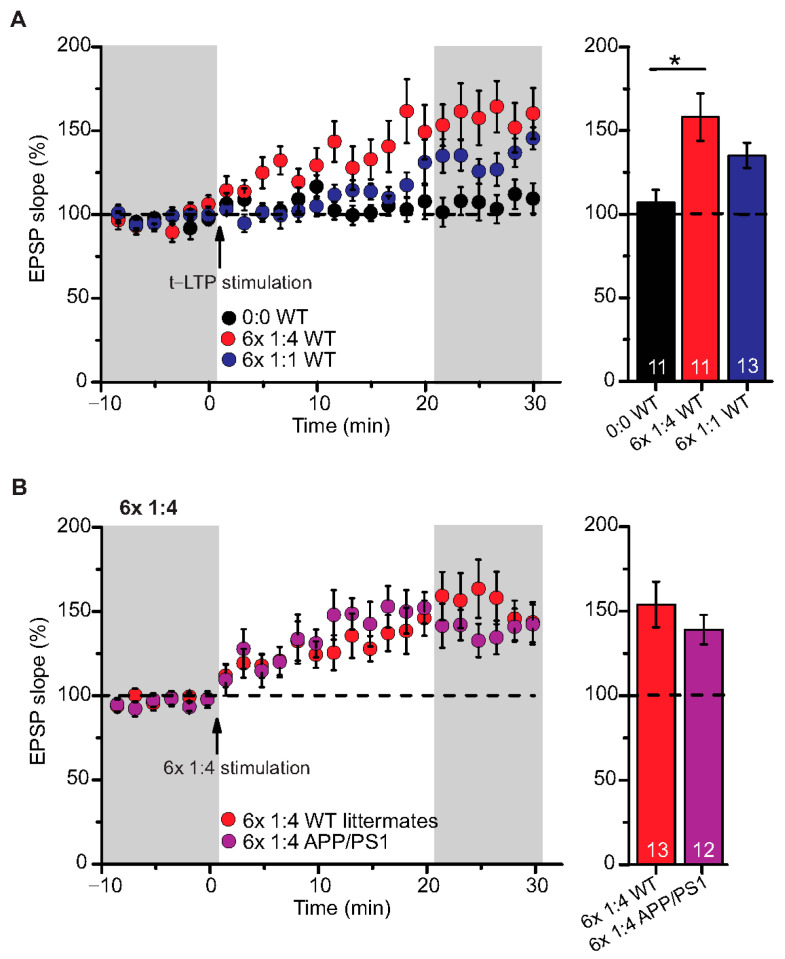
Intact t-LTP in adult APP/PS1 mice induced by 6× 1:4 STDP paradigm. Whole cell patch clamp recording of t-LTP in acute hippocampal slices from 6-month-old wild type and APP/PS1 mice using the STDP paradigms described in Figure 1. Left: Mean time-course of EPSP slopes with either of the two different STDP paradigms (indicated by arrows). Right: Averaged change in EPSP slopes 21–30 min following t-LTP induction normalized to control before t-LTP induction. (**A**) WT mice expressed significant t-LTP induced by 6 × 1:4 stimulation (red circles, n = 11/N = 8; *p* = 0.003), while the 6× 1:1 stimulation induced synaptic change did not yield statistically significant t-LTP (blue circles, n = 13/N = 10; *p* = 0.104) compared to unpaired control (black circles, n = 11/N = 10). (**B**) Hippocampal SC-CA1 synapses in APP/PS1 animals showed unaltered t-LTP (purple circles, n = 12/N = 6; *p* = 0.354), induced by 6× 1:4 stimulation, compared to WT littermate mice (red circles, n = 13/N = 7). Data shown as mean ± SEM. Digits in the bars represent the number of recorded neurons per condition. *: *p* < 0.05, multiple comparisons were performed with ANOVA followed by post hoc Dunnett’s test (**A**) and non-parametric data were compared with Mann–Whitney U-test (**B**).

To the best of our knowledge, STDP has been studied previously exclusively in 4–6 weeks old juvenile or adolescent animals [25,32,33]. To explore STDP properties in adult AD model mice, we first had to establish STDP paradigms that successfully induce t-LTP in adult WT mice. To this aim we investigated in a first series of experiments a low repeat (6× at 0.5 Hz) STDP paradigm that we previously established in juvenile WT mice [34], to induce t-LTP in 6-month-old (adult) C57Bl6/J mice (Figure 1 and Figure 2A).

We used a canonical STDP protocol consisting of 1:1 pairing of presynaptic AP firing followed by firing of a single AP in the postsynaptic CA1 neuron (i.e., 6× 1:1 protocol; Δt = +10 ms), and a 6× 1:4 burst protocol wherein the single postsynaptic AP was replaced by a theta burst of 4 APs fired at a frequency of 200 Hz (Figure 1). The SC-CA1 synapses in 6-month-old WT mice showed significant t-LTP induced by the 6× 1:4 stimulation when compared to non-STDP stimulated negative controls (0:0), while 6× 1:1 stimulation induced synaptic change did not reach statistically significant t-LTP (Figure 2A; 0:0 WT: 106.9 ± 7.7% (n = 11/N = 10); 6× 1:4 WT: 157.9 ± 14.2% (n = 11/N = 8); 6× 1:1 WT: 134.3 ± 7.5% (n = 13/N = 10)).

ANOVA analyses revealed a significant main effect (F_(2,32)_ = 6.142; *p* = 0.0055), and post hoc Dunnett’s test showed *p* = 0.0027 for 0:0 WT vs. 6× 1:4 WT and *p* = 0.1041 for 0:0 WT vs. 6× 1:1 WT. Nevertheless, the 6× 1:1 STDP paradigm induced significant t-LTP in comparison to negative controls as well as its own baseline (two-tailed Student’s t-test t_(22)_ = −2.5518; *p* = 0.0182; one-sample t-test t_(12)_ = 4.595; *p* = 0.0006). While these results revealed that t-LTP can be induced in 6-month-old WT mice with both low repeat STDP paradigms, a higher magnitude of t-LTP was observed in WT animals in response to the 6× 1:4 compared to the 6× 1:1 protocol.

#### Low Repeat STDP Paradigm Induced Timing-Dependent (t-) LTP in Adult APP/PS1 Mice

Next, we tested the 6× 1:4 t-LTP protocol in APP/PS1 mice compared to their WT littermates and observed unaltered t-LTP at SC-CA1 synapses in APP/PS1 animals induced by 6× 1:4 stimulation, compared to WT littermates (Figure 2B; 6× 1:4 WT: 153.8 ± 13.5% (n = 13/N = 7); 6× 1:4 APP/PS1: 138.9 ± 8.7% (n = 12/N = 6), Mann–Whitney U-test U = 87.0, *p* = 0.624). These results reveal that APP/PS1 mice express on average intact t-LTP induced with 6× 1:4 stimulation when the position of Aβ plaques in the slice is not considered.

### 2.2. High Repeat STDP Stimulation Paradigm Induced t-LTP in Adult APP/PS1 Mice

**Figure 3:**
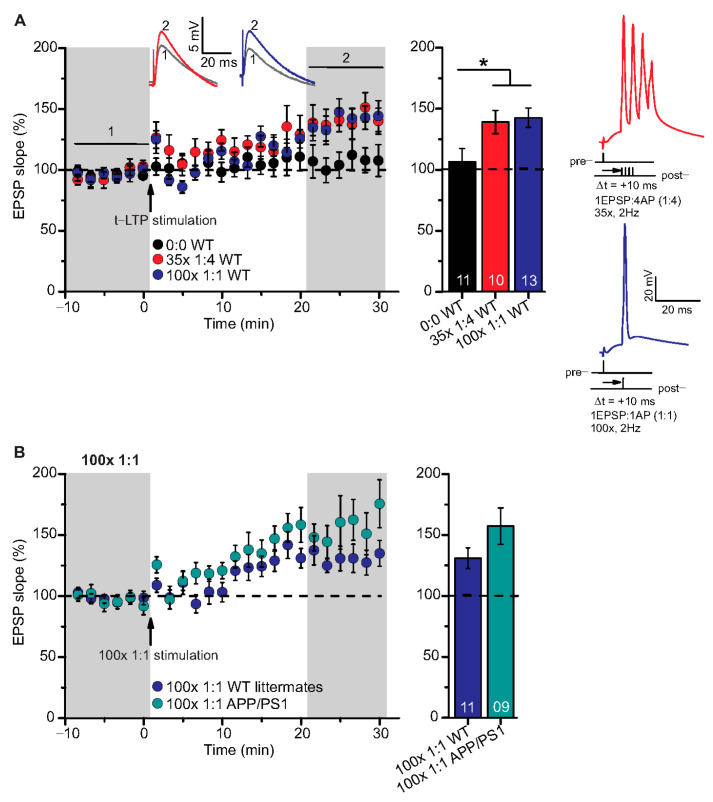
High repeat STDP stimulation paradigm induced t-LTP in adult wild type and APP/PS1 mice. Whole cell patch clamp recordings of t-LTP as described in Figure 2. Left: mean time course for EPSP slopes with either of the two different STDP paradigms (indicated by arrows). Right: Averaged changes in EPSP slopes 21–30 min following t-LTP induction normalized to control before t-LTP induction. (**A**) Insets: average EPSP before (1) and after t-LTP induction (2). 35× 1:4 (red circles, n = 10/N = 10; *p* = 0.04) and 100× 1:1 stimulation (blue circles, n = 13/N = 12; *p* = 0.02) induced significant t-LTP in WT mice in comparison to unpaired control (0:0; black circles, n = 11/N = 7). Insets: 35× 1:4 (red color) and 100× 1:1 (blue color) STDP paradigms at 2 Hz. (**B**) 100× 1:1 stimulation induced comparable t-LTP in APP/PS1 mice (cyan circles, n = 9/N = 5; *p* = 0.126) and WT littermates (blue circles, n = 11/N = 5). Scale bars are shown in the respective insets. Data shown as mean ± SEM. Digits in the bars indicate the number of recorded neurons per condition. *: *p* < 0.05, multiple comparisons were performed with ANOVA followed by post hoc Dunnett’s test (**A**) and parametric data were compared with two-tailed Student’s t-test (**B**).

To further explore STDP in adult 6-month-old WT and APP/PS1 mice, we tested the 1:1 and the 1:4 protocols with higher repeat numbers that were shown previously to elicit t-LTP in juvenile animals (compare [25]). Here, we argued that fully matured SC-CA1 synapses in adult animals might need stronger STDP paradigms for robust t-LTP, and that this t-LTP might be more sensitive to amyloidosis pathology in APP/PS1 than the low repeat protocols. First, we asked whether t-LTP induced with high repeat paradigms (i.e., 35× 1:4 and 100× 1:1) is different in magnitude or time-course compared to t-LTP induced with low repeat paradigms in WT mice (see Figure 3A).

Both, 100× 1:1 and 35× 1:4 stimulation induced significant t-LTP at SC-CA1 synapses in adult WT mice in comparison to unpaired control (Figure 3A; 0:0 WT: 106.3 ± 11.0% (n = 11/N = 7); 35× 1:4 WT: 139.1 ± 9.5% (n = 10/N = 10); 100× 1:1 WT: 142.0 ± 7.9% (n = 13/N = 12)). ANOVA analyses revealed a significant main effect (F_(2,31)_ = 4.386 *p* = 0.0210), and post hoc Dunnett’s test showed *p* = 0.0455 for 0:0 WT vs. 35× 1:4 WT and *p* = 0.0189 for 0:0 WT vs. 100× 1:1 WT. These results reveal that the 100× 1:1 high repeat stimulation protocol yields more effective t-LTP than its low repeat 6× 1:1 t-LTP (compare Figure 2A and Figure 3A). Thus, we next asked whether the 100× 1:1 induced t-LTP might be impaired in APP/PS1 mice. However, we observed no significant difference between t-LTP magnitudes at SC-CA1 synapses in APP/PS1 mice vs. WT littermates induced by 100× 1:1 stimulation (Figure 3B; 100× 1:1 WT: 130.9 ± 8.5% (n = 11/N = 5); 100× 1:1 APP/PS1: 157.3 ± 14.9% (n = 9/N = 5), two-tailed Student’s t-test t_(18)_ = −1.6047 *p* = 0.126).

Together, these findings indicate that t-LTP can be induced successfully with high repeat STDP stimulation paradigms in 6-month-old adult WT mice, and this t-LTP remains on average intact in APP/PS1 animals when the location of Aβ plaques in the slice is not addressed experimentally.

### 2.3. T-LTP Induced with 6× 1:4 Stimulation at SC-CA1 Synapses in Adult APP/PS1 Mice is Impaired Selectively in CA1 Neurons Located Near to Aβ Plaques

**Figure 4:**
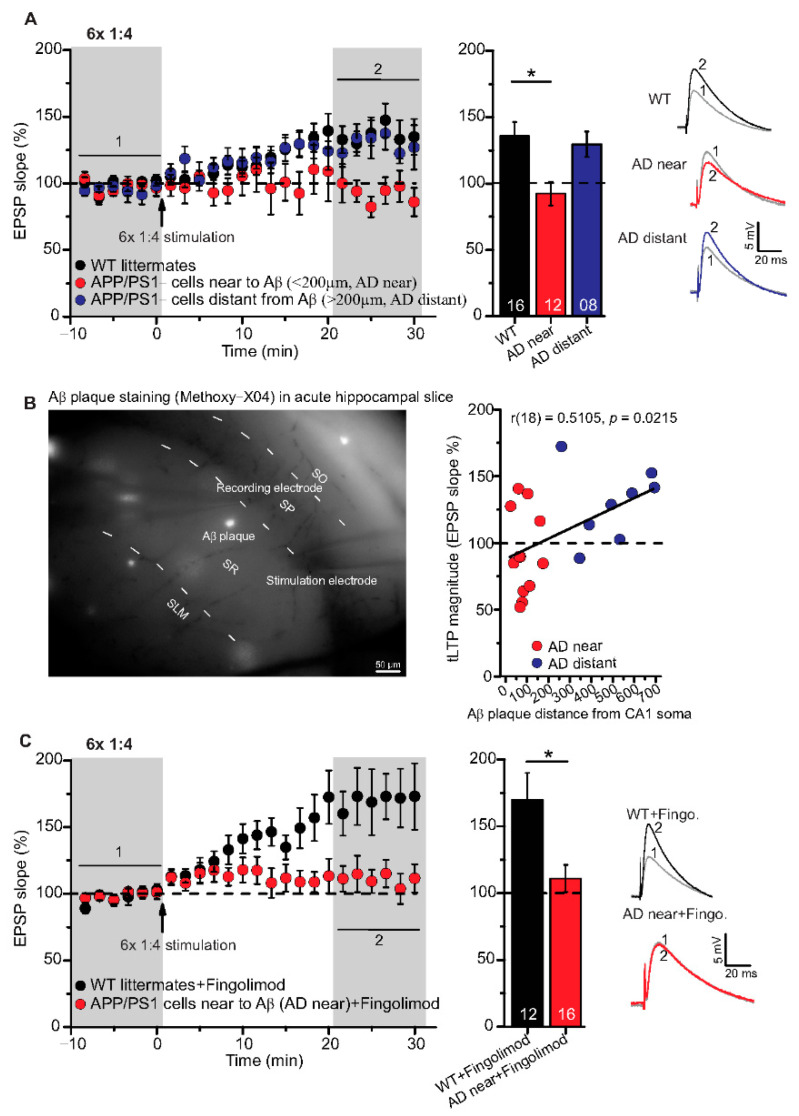
Impairment of t-LTP in adult APP/PS1 mice depends on proximity of recorded CA1 neuron to Aβ plaques. Whole cell patch clamp recording of t-LTP in acute hippocampal slices as described in Figure 2. Left: Mean time-course of EPSP slopes for 6× 1:4 paradigm (indicated by arrow). Right: Averaged change in EPSP slopes 21–30 min following t-LTP induction normalized to control before t-LTP induction. (**A**) APP/PS1 mice showed impaired t-LTP in CA1 neurons near to Aβ plaques (<200 µm, AD near, red circles, n = 12/N = 9; vs. WT: *p* = 0.009), while in CA1 neurons distant from Aβ plaques (>200 µm, AD distant, blue circles, n = 8/N = 4; vs WT: *p* = 0.92), t-LTP magnitude was comparable to WT littermate mice (black circles, n = 16/N = 9). (**B**) Methoxy-X04 staining of Aβ plaques in acute hippocampal slices. APP/PS1 mice showed moderate positive correlation between CA1 cell soma distance from Aβ plaque and t-LTP magnitude (Pearson correlation coefficient r(18) = 0.5105; *p* = 0.02). (**C**) No rescue of impaired t-LTP in APP/PS1 mouse CA1 neurons near to plaques after chronic fingolimod (FTY720) treatment (red circles, n = 16/N = 7; *p* = 0.01), compared to fingolimod treated WT littermates (black circles, n = 12/N = 7). Data are shown as mean ± SEM. Insets: average EPSP before (1) and after t-LTP induction (2). Digits in the bars indicate the number of recorded neurons per condition. *: *p* < 0.05, multiple comparisons were performed with ANOVA followed by post hoc Tukey’s test (**A**), while non-parametric data were compared with Mann–Whitney U-test (**C**). Aβ: amyloid-beta, SO: stratum (S) oriens, SP: S. pyramidale, SR: S. radiatum, SLM: S. lacunosum moleculare.

Although high frequency stimulation induced conventional LTP at SC-CA1 synapses was reported previously to be impaired in 5–6-month-old animals of the same APP/PS1 mouse strain used in the present study [10,35], our above-described results showed that t-LTP tested was not significantly altered at this age. Since we argued that the proximity of Aβ plaques to the recorded CA1 neuron might be decisive to observe significant t-LTP deficits in APP/PS1 animals, we next focused on t-LTP in CA1 neurons in the vicinity of Aβ plaques that we visualized by staining with the dye methoxy-X04 (for staining procedure, compare Methods). Stained Aβ plaques in APP/PS1 mice were visible in stratum radiatum, stratum oriens, and in the pyramidal cell layer of the CA1 area, but at this age (6-month), the density of Aβ plaques is relatively sparse (compare [10,36]). Thus, we decided to record t-LTP selectively in CA1 pyramidal neurons in the vicinity of Aβ plaques. To this aim we determined the distance between the recorded CA1 neuron soma and the nearest plaque. We observed that in CA1 neurons with their somata <200 µm away from the border of the nearest Aβ plaque (AD near), t-LTP induced by 6× 1:4 stimulation was significantly impaired (*p* = 0.0093) in comparison to t-LTP in methoxy-X04 treated WT littermate mice. Moreover, we observed comparable t-LTP magnitude in CA1 neurons >200 µm away from the border of the nearest Aβ plaque (AD distant) relative to methoxy-X04 treated WT littermates (Figure 4A; WT: 135.8 ± 10.6% (n = 16/N = 9); AD near: 92.4 ± 9.1% (n = 12/N = 9); AD distant: 129.6 ± 9.6% (n = 8/N = 4)).

ANOVA analyses showed a significant main effect (F_(2,33)_ = 5.338; *p* = 0.0098), and post hoc Tukey’s test revealed *p* = 0.0093 for WT vs. AD near, *p* = 0.9153 for WT vs. AD distant, and *p* = 0.0762 for AD near vs. AD distant. Interestingly, when plotting t-LTP magnitude of recorded CA1 cells vs. distance to nearest plaque, we observed a moderate positive correlation between both parameters (Figure 4B; Pearson correlation coefficient r(18) = 0.5105, *p* = 0.0215).

Altogether, these results indicate that the proximity of CA1 pyramidal cell somata to Aβ plaques crucially regulates the extent of impairments in t-LTP at the single neuron level. Different STDP paradigms, their characteristics and t-LTP magnitude at SC-CA1 synapses in 6-month-old C57Bl/6J, APP/PS1 mice, and WT littermates are summarized in Table 1.

### 2.4. Chronic Fingolimod Treatment of APP/PS1 Mice Does Not Rescue Impaired t-LTP in CA1 Neurons Located in the Vicinity of Aβ Plaques

The FDA approved anti-multiple sclerosis drug fingolimod (FTY720, sphingosine-1 phosphate receptor modulator) has been shown previously to ameliorate Aβ pathology, as well as associated deficits in synaptic plasticity and memory formation in AD mice [10,37,38]. Based on these evidences, we tested the same regime of 1-month chronic fingolimod treatment (compare with Methods) that we reported previously to rescue AD deficits in APP/PS1 mice (compare [10]) for its potential to rescue 6× 1:4 t-LTP deficits in CA1 neurons near to Aβ plaques. However, this treatment did not rescue 6× 1:4 induced t-LTP at SC-synapses of CA1 neurons with their somata near to plaques (AD near) (Figure 4C; WT + fingolimod: 169.9 ± 20.1% (n = 12/N = 7); AD near + fingolimod: 111.1 ± 10.2% (n = 16/N = 7), Mann–Whitney U-test: U = 42.0, *p* = 0.012). This suggests that chronic fingolimod treatment is not sufficient to rescue the 6× 1:4 induced t-LTP deficits in CA1 pyramidal cells of APP/PS1 mice.

### 2.5. CA1 Pyramidal Neurons in APP/PS1 Animals Showed Comparable Basal Electrical Properties as WT Littermates, Irrespective of Aβ Plaques Location

**Figure 5:**
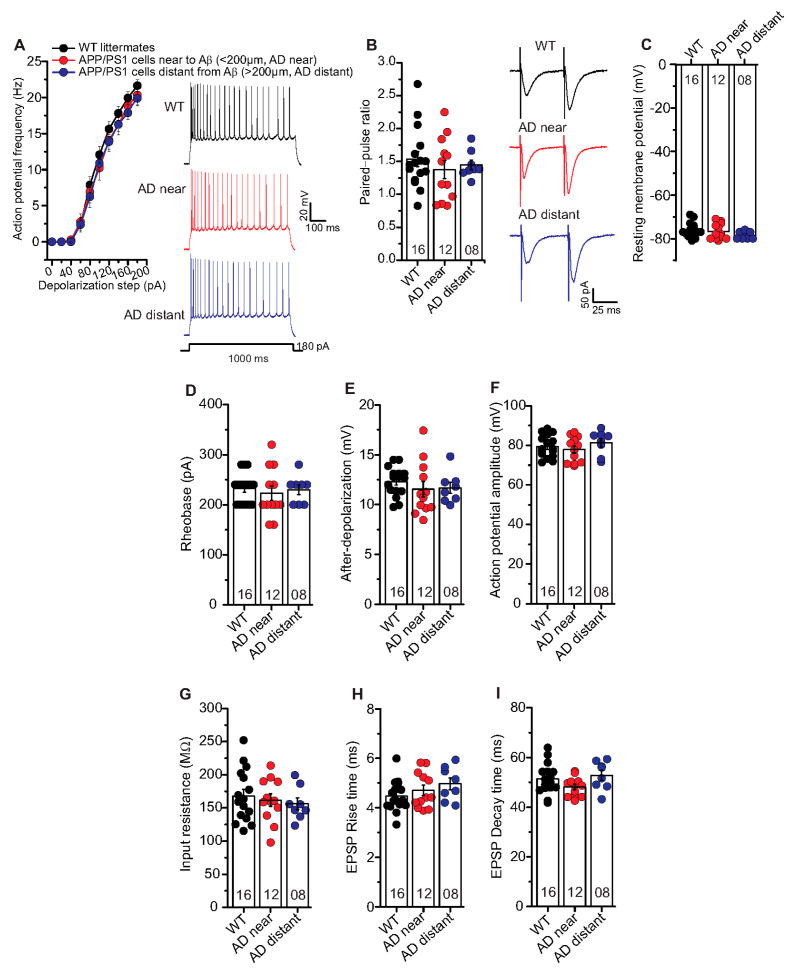
Unaltered basal electrical and synaptic properties of CA1 pyramidal neurons in the vicinity of Aβ plaques in APP/PS1 mice. Whole cell patch clamp recordings (current clamp except for paired-pulse facilitation recorded in voltage clamp) from CA1 neurons in acute hippocampal slices near to Aβ plaques in APP/PS1 mice (<200 µm, AD near, red circles), CA1 cells distant from Aβ plaques in APP/PS1 mice (>200 µm, AD distant, blue circles), and CA1 neurons from WT littermates (black circles). (**A**) The AD near and AD distant groups showed similar intrinsic excitability compared to age-matched WT littermate mice (ANOVA; *p* = 0.07). Insets: action potentials (APs) firing in CA1 neurons in response to 180 pA (for 1000 ms) somatic current injection from WT, AD near and AD distant groups. (**B**) Paired-pulse ratio (PPR) at SC-CA1 synapses in AD near and AD distant group was similar to PPR in WT littermates (*p* = 0.62). Insets: PPR at inter-stimulus interval of 50 ms in CA1 pyramidal cells from WT, AD near and AD distant groups. Hippocampal CA1 neurons from AD near, AD distant group showed similar resting membrane potentials ((**C**); *p* = 0.26), rheobases ((**D**); *p* = 0.81), AP amplitudes ((**E**); *p* = 0.45), after-depolarizations ((**F**); *p* = 0.55), input resistances ((**G**); *p* = 0.73), EPSP rise times ((**H**); *p* = 0.23), and EPSP decay times ((**I**); *p* = 0.14) compared to WT littermates. Digits in the bars represent the number of recorded neurons per condition, at least from three different animals per group. Data displayed as mean ± SEM. Multiple comparisons were performed with ANOVA.

In the aforementioned results, we observed clear deficits in t-LTP at single neuron level in APP/PS1 mice only in CA1 neurons near to Aβ plaques (AD near). Therefore, we investigated whether the AD pathology also affected the basal electrical properties of hippocampal CA1 neurons. First, we focused on general differences between the genotypes irrespective of Aβ plaques location (Supplementary Figure S1). Since these results did not reveal any general differences between hippocampal CA1 neurons from APP/PS1 and WT littermates (Supplementary Figure S1), we investigated whether any such differences were evident when comparing CA1 neurons located near vs. distant to Aβ plaques of APP/PS1 animals. When testing basal electrical and basal synaptic properties of SC inputs to CA1 neurons, we did not observe any significant differences between properties of neurons near plaques (AD near) compared to CA1 cells with their somata distant from plaques (AD distant). Likewise, both groups showed no significant differences in all these parameters compared to WT littermate controls. Thus, AP frequency (Figure 5A: AP frequency in response to a 180 pA depolarizing current step: WT: 21.6 ± 0.9 Hz (n = 16/N = 9); AD near: 20.3 ± 1.4 Hz (n = 12/N = 9); AD distant: 19.9 ± 1.0 Hz (n = 8/N = 4), ANOVA_(2,330)_ = 2.7340; *p* = 0.0664), rheobase (Figure 5D; WT: 232.5 ± 07.5 pA (n = 16/N = 9); AD near: 223.3 ± 14.3 pA (n = 12/N = 9); AD distant: 230.0 ± 10.0 pA (n = 8/N = 4), ANOVA_(2,33)_ = 0.2105; *p* = 0.8113), after-depolarization (Figure 5E; WT: 12.3 ± 0.4 mV (n = 16/N = 9); AD near: 11.5 ± 0.8 mV (n = 12/N = 9); AD distant: 11.7 ± 0.5 mV (n = 8/N = 4), ANOVA_(2,33)_ = 0.5997; *p* = 0.5549), action potential amplitude (Figure 5F; WT: 79.4 ± 1.4 mV (n = 16/N = 9); AD near: 77.9 ± 1.8 mV (n = 12/N = 9); AD distant: 81.4 ± 2.2 mV (n = 8/N = 4), ANOVA_(2,33)_ = 0.8169; *p* = 0.4505) were not significantly different between the groups.

Moreover, PPR at inter-stimulus interval of 50 ms (Figure 5B; WT: 1.54 ± 0.1 (n = 16/N = 9); AD near: 1.38 ± 0.1 (n = 12/N = 9); AD distant: 1.45 ± 0.1 (n = 8/N = 4), ANOVA_(2,33)_ = 0.4812; *p* = 0.6223), EPSP rise times (10–90% Trace Fit; Figure 5H; WT: 4.47 ± 0.16 ms (n = 16/N = 9); AD near: 4.71 ± 0.21 ms (n = 12/N = 9); AD distant: 4.97 ± 0.24 ms (n = 8/N = 4), ANOVA_(2,33)_ = 1.552; *p* = 0.2270), and EPSP decay times (90–10% Trace Fit; Figure 5I; WT: 51.4 ± 1.5 ms (n = 16/N = 9); AD near: 48.2 ± 1.1 ms (n = 12/N = 9); AD distant: 52.8 ± 2.0 ms (n = 8/N = 4 ), ANOVA_(2,33)_ = 2.083; *p* = 0.1407) were comparable between the groups. Further, we observed similar resting membrane potential (Figure 5C; WT: –76.3 ± 0.8 mV (n = 16/N = 9); AD near: 76.8 ± 1.0 mV (n = 12/N = 9); AD distant: -78.5 ± 0.6 mV (n = 8/N = 4), ANOVA_(2,33)_ = 1.387; *p* = 0.2641) and input resistance (Figure 5G; WT: 167.9 ± 9.9 MΩ (n = 16/N = 9); AD near: 161.7 ± 9.5 MΩ (n = 12/N = 9); AD distant: 156.2 ± 8.9 MΩ (n = 8/N = 4), ANOVA_(2,33)_ = 0.3197; *p* = 0.7286) between the groups.

These results revealed that the observed impairment in t-LTP in APP/PS1 mice cannot be accounted for by differences of neuronal excitability or basic synaptic properties in postsynaptic CA1 pyramidal cells of this AD mouse model.

### 2.6. APP/PS1 Mice Show Differences in Spontaneous Excitatory Postsynaptic Currents (EPSCs)

**Figure 6:**
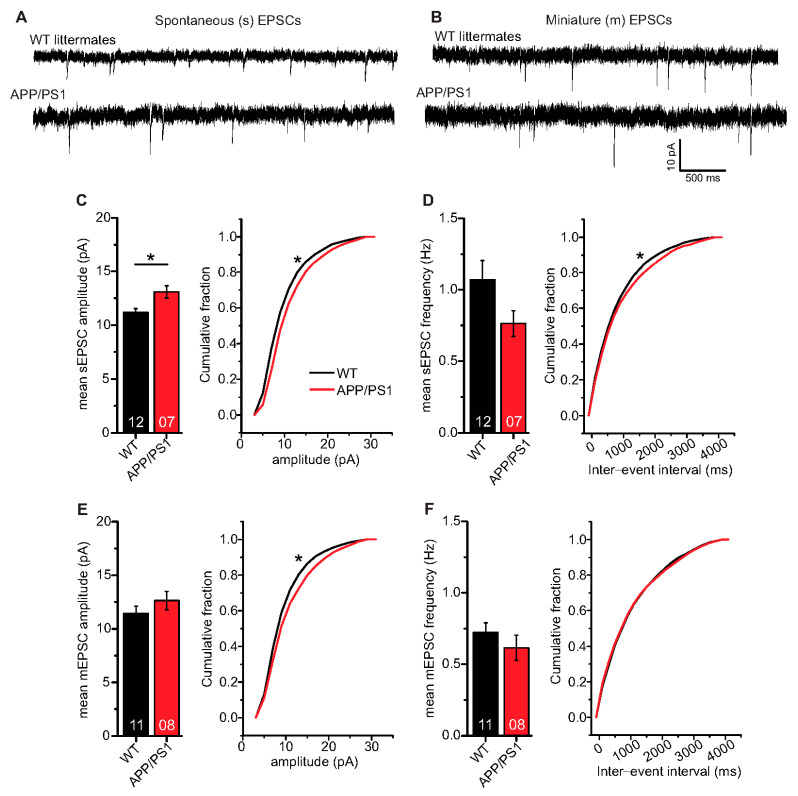
Spontaneous and miniature excitatory synaptic transmission in CA1 neurons of 6-month-old adult APP/PS1 mice. Whole cell patch clamp recordings (voltage clamp) of synaptic currents from CA1 neurons in acute hippocampal slices. APP/PS1 mice specified by red symbols, WT littermate mice represented by black symbols. (**A**,**B**) spontaneous (s) and miniature (m) EPSCs in acute hippocampal slices from WT littermate and APP/PS1 mice, respectively. (**C**) APP/PS1 mice showed significantly higher mean sEPSC amplitudes (*p* = 0.008). Moreover, APP/PS1 mice showed significantly different cumulative sEPSC amplitude distribution (*p* < 0.0001). (**D**) CA1 neurons from APP/PS1 animals displayed comparable mean sEPSC frequencies as WT littermate animals (*p* = 0.11). However, APP/PS1 and WT mice expressed significantly different distribution of sEPSC inter-event intervals (IEIs; *p* = 0.005). (**E**) CA1 neurons from both WT littermate and APP/PS1 mice showed similar mean mEPSC amplitudes (*p* = 0.28), but significantly different cumulative mEPSC amplitude distributions (*p* < 0.0001). (**F**) CA1 neurons from APP/PS1 animals displayed similar mean mEPSC frequencies (*p* = 0.25) and mEPSC IEI distribution as WT littermates (*p* = 0.52). Digits in the bars show the number of recorded neurons per condition, at least from three different animals per group. Data shown as mean ± SEM. *: *p* < 0.05, statistical analyses were performed with two-tailed Student’s t-test (parametric data) and Mann–Whitney U-test (non-parametric data). Cumulative frequency distributions were analyzed with Kolmogorov–Smirnov test.

In a final set of experiments, we were interested to learn whether AP driven spontaneous synaptic network activity or miniature excitatory synaptic transmission differed between APP/PS1 mice and WT littermates. To address this question, we studied spontaneous (s) and miniature (m) excitatory postsynaptic currents (EPSCs) in APP/PS1 mouse hippocampal CA1 neurons and WT littermates. Here, hippocampal CA1 neurons in APP/PS1 mice showed significantly higher mean sEPSC amplitudes (Figure 6C; WT: 11.17 ± 0.37 pA (n = 12/N = 4); APP/PS1: 13.08 ± 0.56 pA (n = 7/N = 3), two-tailed Student’s t-test t_(17)_ = −2.9871 *p* = 0.0083), and a significantly different cumulative sEPSC amplitude distribution compared to WT littermates (Figure 6C; Kolmogorov–Smirnov test D = 0.1290; *p* < 0.0001).

Mean sEPSC frequencies in CA1 neurons were decreased in APP/PS1 mice compared to WT littermates, although this effect did not reach statistical significance (Figure 6D; WT: 1.07 ± 0.14 Hz (n = 12/N = 4); APP/PS1: 0.76 ± 0.09 Hz (n = 7/N = 3), Mann–Whitney U-test U = 61.0, *p* = 0.108), whereas the sEPSC inter-event interval distribution differed beween the two groups (IEIs, Figure 6D; Kolmogorov–Smirnov test D = 0.0518; *p* = 0.0046). When synaptic events were recorded in the presence of 1 µM tetrodotoxin (TTX) to block firing of APs, recorded mEPSCs, as a measure of quantal release probabilities showed comparable mean mEPSC amplitudes (Figure 6E; WT: 11.43 ± 0.68 pA (n = 11/N = 4); APP/PS1: 12.64 ± 0.86 pA (n = 8/N = 3), two-tailed Student’s t-test t_(17)_ = −1.1133 *p* = 0.2811), while significantly different cumulative mEPSC amplitudes were evident (Figure 6E; Kolmogorov–Smirnov test D = 0.0843; p<0.0001). Moreover, we observed similar mean mEPSC frequencies (Figure 6F; WT: 0.72 ± 0.07 Hz (n = 11/N = 4); APP/PS1: 0.62 ± 0.09 Hz (n = 8/N = 3), Mann–Whitney U-test U = 58.0, *p* = 0.248) and comparable IEI distribution of mEPSC in WT and APP/PS1 animals (Figure 6F; Kolmogorov–Smirnov test D = 0.0256; *p* = 0.5174).

Taken together, the spontaneous and miniature EPSC analyses in CA1 neurons revealed that in APP/PS1 mice, sEPSC amplitudes are increased whereas sEPSC frequencies are decreased, although this effect did not reach statistical significance, compared to WT littermates. On the other hand, APP/PS1 and WT littermate mice express comparable mEPSC amplitudes and frequencies. Furthermore, sIPSCs (inhibitory postsynaptic currents) are slightly decreased in amplitude and frequency in APP/PS1 mice compared to WT littermates, but also this effect did not reach statistical significance. Moreover, mIPSC properties are indistinguishable between genotypes (see Supplementary Figure S2 for IPSCs).

## 3. Discussion

High frequency stimulation (HFS) induced long-term potentiation (LTP) represents a traditional correlation-based type of synaptic plasticity. In contrast, spike timing-dependent plasticity (STDP) captures the importance of causality in determining the direction of synaptic modification [39]. Moreover, STDP has rapidly gained interest, because of its combination of simplicity, physiological plausibility, and computational power. Nonetheless, the current knowledge of molecular and cellular mechanisms of STDP and its role in pathophysiology is scarce. While numerous previous studies reported deficits in HFS induced LTP in the hippocampus as an early event in Alzheimer’s disease (AD; [40,41,42,43,44]), the impact of AD on STDP is unknown. In the current study, we therefore assessed timing-dependent (t-) LTP at hippocampal Schaffer collateral (SC)—cornu ammonis (CA) 1 synapses in slices of 6-month-old APP/PS1 mice, which serve as a β-amyloidosis mouse model. Using patch clamp recordings, we were able to identify STDP paradigms that successfully induced t-LTP at SC-CA1 synapses in fully matured 6-month-old adult WT mice. The 6× 1:4 stimulation induced t-LTP was not significantly impaired in 6-month-old APP/PS1 mice, under conditions when proximity of amyloid beta (Aβ) plaque to the recorded neurons was not resolved. However, this 6× 1:4 t-LTP was strongly impaired if the nearest Aβ plaque was <200 µm away from the recorded neuron, and was not restored by chronic treatment of APP/PS1 mice with the anti-inflammatory drug Fingolimod (FTY720).

### 3.1. Timing-Dependent LTP in 6-Month-Old Adult WT Mice

While t-LTP is typically studied in juvenile animals, it was not previously reported to exist in 6-month-old WT mice (e.g., [25,32,33,45,46]). The results of the present study reveal that t-LTP can be induced at SC-CA1 synapses in acute hippocampal slices of 6-month-old mice with a 1:1 (canonical) and a 1:4 (burst) protocol that mimics postsynaptic theta bursts of action potentials (APs)—a firing pattern that is observed during learning processes in vivo [47]. Importantly, 6 pairings of pre- and postsynaptic APs at low frequency (0.5 Hz) were sufficient to induce t-LTP (Figure 2A). Interestingly, while also high repeat numbers of STDP stimulation (i.e., 100× 1:1 and 35× 1:4 protocols at 2 Hz) successfully induced t-LTP; these stronger protocols were neither more successful nor induced a higher magnitude of t-LTP (compare Figure 2A and Figure 3A). Since this mild physiologically relevant stimulation with 6× 1:1 or 6× 1:4 paradigms induced t-LTP in adult mice (this study), but are equally effective also in 1 month old juvenile mice [34], our results indicate that physiological maturation does not affect STDP in the hippocampus of normal control mice.

### 3.2. Intact Timing-Dependent LTP in Adult APP/PS1 Mice When Aβ Plaque Location Was Not Resolved

In 6-month-old APP/PS1 mice t-LTP elicited by either 6× 1:4 or 100× 1:1 stimulation was intact (Figure 2B and Figure 3B). However, as an early event in AD mouse models, synaptic dysfunctions such as alterations in conventional HFS induced LTP of hippocampal synaptic transmission have been observed by several earlier studies [40,41,42,43,44]. Importantly, these previous studies used extracellular field potential recordings that measure the summed synaptic responses of a larger population of neurons. In contrast, STDP is measured at the single neuron level. Furthermore, all studies investigating HFS induced LTP (field potential measurements) typically employ high frequency/theta burst stimulations for LTP induction under conditions of intact GABAergic inhibition, while pairing of presynaptic and postsynaptic APs used for t-LTP induction in the current study is typically recorded in the presence of GABA_A_ antagonists (see Methods; [25,48,49]). These differences in LTP induction can have great influence on the recruitment of signaling cascades for expression of LTP compared to t-LTP, and this could account for intact t-LTP but impaired HFS induced LTP in 6-month-old APP/PS1 mice.

### 3.3. Impaired t-LTP in CA1 Neurons Located in the Vicinity of Aβ Plaques in Adult APP/PS1 Mice

Another explanation for the intact 6× 1:4 t-LTP in APP/PS1 mice could be that recorded single CA1 neurons might not be located near enough to Aβ plaques. Importantly, given the relatively low density of Aβ plaques in 6-month-old APP/PS1 hippocampus (see Figure 4B; compare [10]), it is likely that we recorded from CA1 pyramidal neurons farther away from a plaque if the location of the nearest plaque was not determined. Since we argued that the density or location of Aβ plaques might be decisive to observe clear t-LTP deficits in APP/PS1 mice, we next investigated t-LTP in CA1 pyramidal neurons in the proximity of Aβ plaques. APP/PS1 mice showed clearly impaired t-LTP in CA1 cells near to Aβ plaques (<200 µm, Figure 4A). This indicates that the proximity of Aβ plaques is indeed decisive to observe t-LTP deficits under our recording conditions. Although the Aβ plaques were identified as a principle component in AD histopathology a century ago, the pathogenic mechanisms of Aβ plaques are still unclear (see reviews [30,50,51,52,53]. However, our results suggest that Aβ plaques and most likely associated soluble Aβ species, which are present at higher concentration around plaques, interfere with t-LTP, thereby contributing to cognitive decline. In this respect it is important to note that in CA1 pyramidal neurons farther away from Aβ plaques in APP/PS1 mice (>200 µm, Figure 4A), t-LTP was unaltered and comparable to t-LTP in WT littermates. Furthermore, we observed a dependence of t-LTP magnitude from plaque distance (Figure 4B), which further supports an important role of plaque-associated toxicity for t-LTP. However, the detailed mechanism(s) by which Aβ plaques disrupt t-LTP at SC-CA1 synapses remains to be investigated.

### 3.4. Chronic Fingolimod Treatment Did Not Rescue Impaired t-LTP in APP/PS1 Mice

Treatment with fingolimod (for 1-month) alone was not sufficient to rescue t-LTP in adult APP/PS1 mice (Figure 4C). It has been described that some neuroprotective effects of fingolimod are mediated through the elevation of brain-derived neurotrophic factor (BDNF) expression [38,54,55] and through reduced neuroinflammation (reviewed in [7,56]). Interestingly, microglia are involved in neuroinflammation and has been reported to affect synaptic remodeling during activity-dependent synaptic plasticity (LTP) in the healthy adult brain (see review [57,58]). Moreover, Nazari and colleagues described a fingolimod mediated rescue of impaired conventional LTP after stroke [59], while others reported fingolimod induced rescue of correlation based synaptic plasticity (LTP) in mouse models of Huntington’s and Alzheimer’s disease [10,60]. While the former study suggests an ameliorating effect of fingolimod at the neuronal network level, our current study reveals no rescue of 6× 1:4 induced impaired t-LTP in the proximity of Aβ plaques in adult APP/PS1 mice. These disparate findings might indicate differences in signaling cascades involved in conventional LTP, an associative activity among larger groups of cells, compared to t-LTP, which relies on plasticity at the single cell level.

### 3.5. Unaltered Basal Properties of CA1 Neurons in APP/PS1 Mice

Since we observed t-LTP deficits only in the proximity of Aβ plaques in APP/PS1 mice, we also studied basal electrical properties of CA1 pyramidal neurons in plaque distance-dependent manner. The studied basal electrical properties of the hippocampal CA1 neurons, e.g., intrinsic excitability, paired-pulse facilitation, resting membrane potential and action potential amplitude were intact in APP/PS1 mice (see Figure 5 and Supplementary Figure S1). These observations are consistent with findings from earlier studies [61,62]. Although APP/PS1 mice displayed significantly higher mean EPSC amplitudes and a different cumulative distribution of EPSC amplitudes and inter-event intervals (IEI) for spontaneous EPSCs, we observed largely unaltered properties of miniature EPSC, indicating no gross changes in quantal synaptic transmission at hippocampal SC-CA1 synapses in APP/PS1 mice (EPSCs, Figure 6). In addition, CA1 pyramidal neurons in APP/PS1 mice expressed comparable non-evoked inhibitory synaptic transmission as WT littermate mice (IPSCs, Supplementary Figure S2). Overall, these data indicate that overexpression of APP and PS1 mutations does not generally compromise CA1 pyramidal neurons’ neuronal excitability and basal synaptic transmission in 6-month-old APP/PS1 mice.

While our study is the first to illustrate t-LTP deficits in APP/PS1 mice in an Aβ plaque distance-dependent manner, the molecular and cellular mechanisms underlying this deficit remain to be elucidated in future studies.

## 4. Conclusions

Our study reveals that in 6-month-old wild type mice t-LTP is elicited with similar efficiency by robust high repeat (i.e., 35× 1:4 and 100× 1:1) and more physiological low repeat (i.e., 6× 1:1 and 6× 1:4) STDP protocols. In 6-month-old APP/PS1 mice that show on average a low level of Aβ plaques in CA1 [10,31], these protocols show unaltered t-LTP, although high frequency stimulation (HFS)-induced SC-CA1 LTP measured with field potential recordings is already impaired at this age [10]. However, if patch clamp recorded CA1 neurons are specifically selected for their proximity to an Aβ plaque (Figure 4), they show impaired t-LTP, suggesting that plaque associated molecules block this potentiation. Together, these findings indicate that field potential HFS-LTP is more sensitive to Aβ pathology than t-LTP in single cell recordings, thereby suggesting that the underlying cellular signaling cascades of t-LTP and HFS-LTP differ. This might also explain why HFS-LTP in APP/PS1 mice can be rescued by Fingolimod [10], while the same treatment does not rescue t-LTP (current study). Future studies in aged APP/PS1 mice are required to resolve the full capacity of Fingolimod to rescue synaptic deficits in AD mice.

## 5. Materials and Methods

### 5.1. Animals

For spike timing-dependent plasticity (STDP) experiments, 6-month-old male C57Bl/6J mice (Charles River, Sulzfeld, Germany), amyloid precursor protein/presenilin 1 (APP/PS1) double transgenic mice [31] and their WT littermates, derived from our own breeding colony were used. APP/PS1 mice co-express KM670/671NL mutated amyloid precursor protein (APP) and L166P mutated presenilin (PS) 1 under the control of a neuron-specific Thy1 promoter element. These APP/PS1 animals are well suited for studying the pathomechanism of amyloidosis, a hallmark of Alzheimer’s disease (AD) pathology. The animals were group housed in Makrolon cages at a temperature of 21 ± 2 °C and 12:12 h light/dark cycle, lights on at 07:00 am. They had free access to food and water. Genotypes of APP/PS1 and their WT littermates were determined by PCR from ear punches before the STDP experiments and verified again by tail biopsies after performing and analyzing the experiments. All experimental procedures were performed during the light period of the animals, were in accordance with the ethical guidelines for the use of animals in experiments of the European Committees Council Directive (2010/63/EU), and were approved by the local animal care committee (Landesverwaltungsamt Saxony-Anhalt, approval numbers: IPHY/G/01-1383/16 and IPHY/G/01-1492/18).

### 5.2. Hippocampal Slice Preparation and Maintenance

The animal was anesthetized using isoflurane (Isofluran CP, cp-pharma, Burgdorf, Germany). The loss of consciousness was confirmed by the absence of reflex activity following a toe pinch. The mouse was decapitated immediately, and the brain was separated from the skull. The brain was kept in an ice-cold artificial cerebrospinal fluid (aCSF) containing (in mM): 125 NaCl, 2.5 KCl, 26 NaHCO_3_, 0.8 NaH_2_PO_4_, 25 glucose, 6 MgCl_2_, 1 CaCl_2_, saturated with 95% O_2_ and 5% CO_2_ (pH 7.2–7.4; 303–306 mOsmol/L, Fiske Micro-osmometer Model 210, Fiske associates, Waterford, PA, USA). The cerebellum, brainstem and one 3rd of the frontal brain were removed before brain slicing. Moreover, the ventral part of the brain was cut transversely at an angle of 11° in order to obtain transversal slices. The brain was cut with a vibratome (LEICA VT1200S VIBRATOME, Leica Biosystems, Nuβloch, Germany) and 350 µm thick acute hippocampal slices were collected starting from the first slice that allowed identifying clearly separated dentate gyrus and all cornu ammonis (CA) regions. These slices were used for STDP experiments and represent the intermediate and ventral region of the hippocampus. After slicing, the CA1 region was isolated from excessive CA3 input by a single cut between CA3 and CA2 to reduce spontaneous excitatory post-synaptic potentials (EPSPs). In all STDP recordings, synaptic inhibition was blocked with γ-aminobutyric acid (GABA)_A_ receptor antagonist picrotoxin (100 µM). About 6 slices were transferred into the interface-style chamber and allowed for 25 min to incubate in continuously carboxygenated (5% CO_2_, 95% O_2_) pre-warmed aCSF (200 mL, same composition as slice preparation medium mentioned above) at 34–35 °C to allow the slice surface to recover from blade trauma, followed by at least one hour of recovery at room temperature. All slices were maintained in this interface chamber at room temperature until being transferred to the recording chamber of an upright microscope for electrophysiological recording.

### 5.3. Aβ Plaque Staining with Methoxy-X04 in Acute Hippocampal Slices

The blue fluorescent dye 1,4-bis-(4′-hydroxystyryl)-2-methoxybenzene (methoxy-X04; Tocris, Wiesbaden, Germany) has been used previously as a live stain for Aβ plaques in AD mice [10,36,63,64]. To localize Aβ plaques in recorded hippocampal slices, APP/PS1 mice were injected intraperitoneally 24 h before slice preparation with methoxy-X04 (dissolved in dimethyl sulfoxide (DMSO; Sigma-Aldrich, Taufkirchen, Germany); stock solution: 10 mg/mL) at a concentration of 25 mg/kg body weight. After slice preparation on the day of t-LTP measurement and transfer of a recorded slice into the recording chamber of an upright fluorescence microscope (Zeiss Examiner A1, Carl Zeiss Microscopy GmbH, Jena, Germany), Aβ plaques were visualized using a 10× and 63× water immersion objectives. Fluorescence of methoxy-X04 was excited with a band pass filter (352–402 nm) and the emitted light was passed through a filter cube allowing to selectively detect blue fluorescence (dichroic mirror: 409 nm; bandpass filter: 417–477 nm). Live cell imaging of blue fluorescent Aβ plaques in CA1 area and associated stratum radiatum was performed with a digital CCD camera Photometrics CoolSNAP ES^2^ (Visitron Systems GmbH, Puchheim, Germany). Using a digitized micromanipulator system (Luigs and Neumann SM-5), the distance (x,y,z coordinates) between the recorded CA1 neuron soma and the border of the nearest Aβ plaque was determined. The diagonal distance (compare Figure 4) of Aβ plaque from the CA1 neuron soma was calculated offline with the Pythagorean equation.

### 5.4. Chronic Fingolimod Treatment of APP/PS1 Mice

As a therapeutic strategy to rescue AD related deficits in t-LTP, we tested chronic fingolimod (Abcam, Cambridge, UK) treatment in adult APP/PS1 mice [38,54]. APP/PS1 animals and their WT littermates were injected intraperitoneally (i.p.) with a dose of 1 mg fingolimod/kg body weight [38], every second day for a month. Fingolimod stock solution (200 mM in DMSO) was diluted with 0.9% saline and was stored in aliquots at −20 °C until use. On the day of administration, fingolimod solution was thawed at room temperature, and warmed in 37 °C water bath for 3–4 min before intraperitoneal injection.

### 5.5. Electrophysiology in Acute Hippocampal Slices

For all experiments, 350 μm thick acute, transversal hippocampal slices were used. For whole-cell recordings, pyramidal neurons in CA1 region of hippocampus were visualized with differential interference contrast infrared video microscopy (VX45 Optronis camera, Optronis GmbH, Kehl, Germany; Zeiss Examiner A1 microscope, Carl Zeiss Microscopy, Jena, Germany). For all STDP recordings, 100 µM picrotoxin (Sigma Aldrich, Taufkirchen, Germany, dissolved in ethanol) was added to the extracellular aCSF solution to block GABA_A_ receptors. ACSF was composed of (in mM): 125 NaCl, 2.5 KCl, 25 NaHCO_3_, 0.8 NaH_2_PO_4_, 25 glucose, 2 CaCl_2_, 1 MgCl_2_, saturated with 95% O_2_ and 5% CO_2_ (pH 7.2–7.4; 301–304 mOsmol/L). Slices were incubated for 5–10 min in the recording chamber before start of recording. Whole-cell recordings were performed at 28–31 °C (aCSF perfusion rate, 1.8 mL/min), with glass pipettes (pipette resistance, 4–6 MΩ) filled with intracellular solution containing (in mM): 140 potassium gluconate, 10 HEPES, 20 KCl, 4 Mg-ATP, 0.3 Na-GTP, 10 Na-phosphocreatine; pH was adjusted to 7.2–7.4 using 1 M KOH (280–290 mOsmol/L). The liquid junction potential of +10 mV that was observed before seal formation was corrected. Pipette capacitance, CA1 pyramidal cell capacitance and series resistance were compensated with EPC8 patch clamp amplifier in all voltage clamp and current clamp recordings. Cells were held in current or voltage clamp mode at –70 mV for different experimental approaches. For stimulation of presynaptic Schaffer collaterals (SC; stimulus duration 0.3 ms), a glass pipette (0.7–0.9 MΩ) filled with intracellular solution was placed in stratum radiatum where SC axon bundles are located in the CA1 region. During control and test periods, EPSPs were evoked at 0.05 Hz in current clamp mode. Presynaptic stimulation which caused postsynaptic firing of action potential (AP) or the maximum EPSP amplitude observed was regarded as maximum stimulus intensity. Stimulus strength (µA) was adjusted to evoke 30–50% of maximal stimulation as optimal stimulation intensity and kept constant during the whole experiment. Input resistance was determined by injecting a 20 pA hyperpolarizing current through the recording electrode for a duration of 250 ms; 200 ms prior to each SC stimulation. Cells were accepted for analysis only if the resting membrane potential was between −50 and −70 mV at the start of the recording. Data were discarded if input resistance changed ±30 % throughout the recording or in case of a clear run-up or run-down of EPSP slopes during the first 10 min of a recording. The paired-pulse facilitation (PPF) was tested at 50 ms inter-stimulus interval (ISI) repeated 3 times at 0.05 Hz in voltage clamp mode (holding potential: −70 mV).

Timing-dependent LTP was induced by repeated pairings of an EPSP induced by single presynaptic stimulation evoked by stimulation of SC input and one or four postsynaptic APs induced by one or four somatic current injections (2 ms, 1 nA) via the recording electrode. Different numbers of pairings were used depending on the stimulation protocol as mentioned in the results. T-LTP was induced by causal (pre-post) pairings at positive spike timings. Paradigms using either a 1 EPSP/1 AP (indicated as 1:1) or a 1 EPSP/4 AP (specified as 1:4) sequence were used. For each recorded cell, positive spike timing (i.e., Δt in ms) was determined between the onset of the EPSP and the peak of the first AP. As a (negative) control, recordings with ongoing synaptic stimulation for 40 min at 0.05 Hz, but without pairing with postsynaptic APs, were performed (indicated as 0:0).

### 5.6. Data Acquisition and Data Analyses

Whole-cell recordings were performed using an EPC8 patch clamp amplifier connected to a LiH8+8 interface (HEKA Elektronik, Lambrecht, Germany) and acquired with Patchmaster software (HEKA, Germany). Data were filtered at 3 kHz and digitized at 10 kHz. Data analyses were performed using FitMaster (HEKA, Germany). Synaptic signals were recorded in current clamp mode as EPSP, except for PPF, which was recorded in voltage clamp as excitatory postsynaptic currents (EPSCs). With the help of FitMaster, initial EPSP slopes (i.e., first 2–3 ms after EPSP onset) were determined. In control and t-LTP experiments, EPSP slopes were normalized to the respective mean baseline recorded during the first 10 min prior to STDP stimulation, which was set to 100%. In all experiments, magnitude of synaptic changes (e.g., t-LTP) was determined as the normalized change in mean response size during the last 10 min of measurement (between 21–30 min after t-LTP induction). Further, the numbers of APs fired by a CA1 neuron, in response to different depolarizing somatic current injections (0–180 pA for 1000 ms, 20 pA increments) through the recording electrode, were determined to analyze intrinsic excitability. As another read-out of intrinsic excitability, the stimulation current (depolarization step (10 ms) of 0–400 pA, 40 pA increment) required to elicit one AP in the recorded CA1 cell [65,66] was determined as rheobase. The PPF was estimated by the paired pulse ratio (PPR), calculated as the normalized change in mean response size of the 2nd divided by the 1st EPSC. AP peak amplitude and after-depolarization were measured using FitMaster (demonstrated in supplementary Figure S1E). Similarly to the EPSP slopes, the input resistance was normalized to the respective mean baseline during the first 10 min of recording, which was then set to 100%. Data were discarded if input resistance changed ±30% throughout the STDP measurement. Further, EPSP rise time and decay time was calculated using the Trace Fit function (low level-high level: 10–90%) for the 10 min long baseline in FitMaster.

### 5.7. Whole Cell EPSC Recordings

Spontaneous and miniature EPSCs were studied in hippocampal CA1 pyramidal neurons in acute slices with no CA3-CA1 cut, prepared from APP/PS1 and WT littermate mice brains as described above. The EPSCs were recorded with the intracellular solution containing (in mM): 140 potassium gluconate, 10 HEPES, 20 KCl, 4 Mg-ATP, 0.3 Na-GTP, 10 Na-phosphocreatine; pH was adjusted to 7.2–7.4 using 1 M KOH (280–290 mOsmol/L; [25]). Spontaneous EPSCs were studied in the presence of bicuculine (10 µM, Sigma Aldrich) and miniature EPSCs were examined in the presence of bicuculine (10 µM) and tetrodotoxin (TTX, 1 µM, Tocris) in the extracellular aCSF [67]. EPSCs were recorded at -70 mV holding potential, for the period of 10 min; 250 events from each sweep were used for data analyses. The temperature of external solution in the recording chamber was maintained at 34–36 °C (aCSF perfusion rate- 2.4 mL/min) for EPSC recordings. Data were analyzed manually using the ‘Mini Analysis program’ (version 6.0.7, Synaptosoft, Decatur, GA, USA).

### 5.8. Statistics

Data are specified as mean ± standard error of mean (SEM), and experiments were combined from at least three different animals per group. Furthermore, the number of experiments (n) and number of animals (N) are indicated in the respective parts of the Results. Group size of 10 was determined by power analysis (G*Power, Heinrich-Heine University Düsseldorf, Germany). The distribution of variables was determined with the Shapiro–Wilk test. Statistical analyses were performed with one-sample t-test, paired or unpaired two-tailed Student’s t-test, as applicable. Non-parametric data were analyzed by Mann–Whitney U-test. Multiple comparisons were performed with ANOVA and post hoc Dunnett’s or Tukey’s test. Pearson correlation coefficient was used to determine correlation between distance of CA1 cell from Aβ plaques and t-LTP magnitude. The Kolmogorov-Smirnov test was used to determine significance for cumulative distributions data (E(I)PSCs). Significance levels are designated by *: *p* < 0.05. The statistical procedures used in each experiment are mentioned in the respective text passages. All data were analyzed using Origin 8.1G (Additive GmbH, Diemelstadt, Germany), GraphPad Prism 8 (GraphPad Software, La Jolla, CA, USA), Mystat 12 (Systat, San Jose, CA, USA).

## Figures and Tables

**Table 1:** Summary of different STDP paradigms induced t-LTP at the hippocampal SC-CA1 synapses in 6-month-old C57Bl/6J and APP/PS1 mice.

STDP Paradigm (Presyn. Stim.: Postsyn. AP)	No. of Repeats, Frequency	Normalized t-LTP Magnitude (EPSP Slope %)		
		C57Bl/6J Mice	WT Littermates	APP/PS1 Mice
0:0 (negative control)		106.9 ± 7.7% (n = 11/N = 10)		
1 EPSP:4 AP	6×, 0.5 Hz	157.9 ± 14.2% (n = 11/N = 8) *^,#^	153.8 ± 13.5% (n = 13/N = 7) *	138.9 ± 8.7% (n = 12/N = 6) *
1 EPSP:1 AP	6×, 0.5 Hz	134.3 ± 7.5% (n = 13/N = 10) *		
1 EPSP:4 AP	35×, 2 Hz	139.1 ± 9.5% (n = 10/N = 10) *^#^		
1 EPSP:1 AP	100×, 2 Hz	142.0 ± 7.9% (n = 13/N = 12) *^#^	130.9 ± 8.5% (n = 11/N = 5) *	157.3 ± 14.9% (n = 9/N = 5) *
		**Aβ Plaque Staining (Methoxy-X04)**		
		WT littermates	APP/PS1, cells near to Aβ (<200 µm)	APP/PS1, cells distant from Aβ (>200 µm)
1 EPSP:4 AP	6×, 0.5 Hz	135.8 ± 10.6% (n = 16/N = 9) *	92.4 ± 9.1% (n = 12/N = 9) ^#^	129.6 ± 9.6% (n = 8/N = 4) *
		**Chronic Fingolimod Treatment of APP/PS1 Mice**		
		WT littermates + fingolimod	AD near + Fingolimod	
1 EPSP:4 AP	6×, 0.5 Hz	169.9 ± 20.1% (n = 12/N = 7) *	111.1 ± 10.2% (n = 16/N = 7)	

n: number of experiments/slices, N: number of animals, * *p* < 0.05, one-sample t-test (significant t-LTP compared to its own baseline); ^#^ *p* < 0.05, ANOVA 6× 1:4 vs. 0:0 WT; 35× 1:4 vs. 0:0 WT, 100× 1:1 vs. 0:0 WT; APP/PS1 near Aβ vs. WT littermates.

## Data Availability

The data presented in this study are available on request from the corresponding author. The data are not publicly available due to privacy.

## Supplementary Materials

The following are available online at https://www.mdpi.com/1422-0067/22/3/1378/s1, Figure S1: Intact basal electrical properties of CA1 pyramidal neurons in adult APP/PS1 mice, Figure S2: Spontaneous inhibitory synaptic transmission in adult APP/PS1 mice.
