# Supplementary material for: Impairment of Spike-Timing-Dependent Plasticity at Schaffer Collateral-CA1 Synapses in Adult APP/PS1 Mice Depends on Proximity of Aβ Plaques
Source: Int J Mol Sci. 2021 Jan 30;22(3):1378. doi: 10.3390/ijms22031378 (PMC7866519; doi:10.3390/ijms22031378)
Supplement: Supplementary file 1 [file ijms-22-01378-s001.pdf]

Impairment of spike-timing-dependent plasticity at Schaffer collateral-CA1 synapses in adult APP/PS1 mice depends on proximity of Aβ plaques

Machhindra Garad, Elke Edelmann, Volkmar Leßmann

Supplementary Figures and Methods

Figure 1_Garad

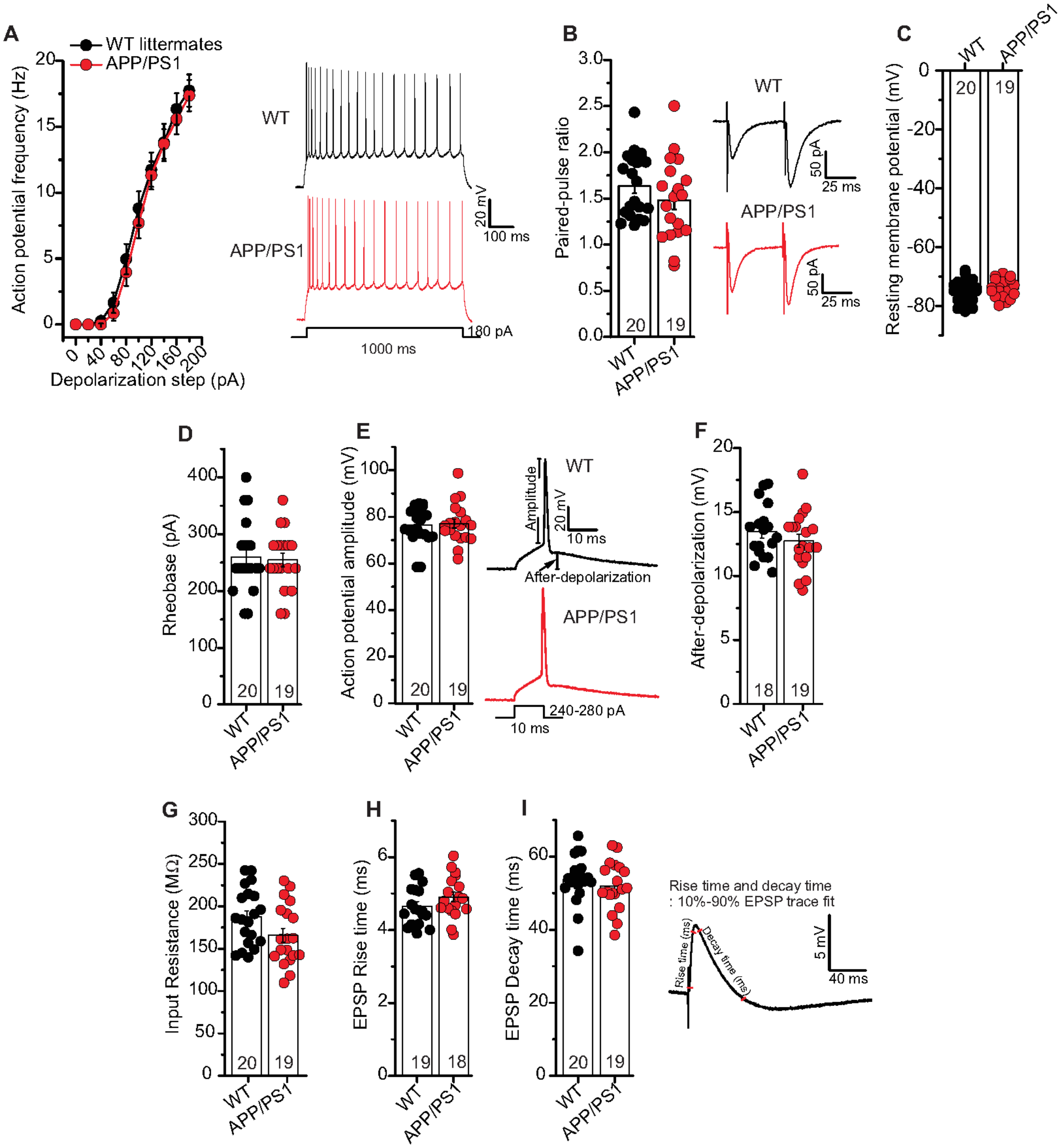

Figure S1: Intact basal electrical properties of CA1 pyramidal neurons in adult APP/PS1 mice. APP/PS1 mice specified by red circles, while WT mice represented by black circles. (**A**) CA1 pyramidal neurons in APP/PS1 mice displayed similar intrinsic excitability as WT littermates (ANOVA $p = 0.67$). Insets: action potentials (APs) firing in CA1 neurons in response to 180 pA (for 1000 ms) somatic current injection from WT littermate and APP/PS1 mice. (**B**) Paired-pulse ratio (PPR) at SC-CA1 synapses in APP/PS1 animals was comparable to WT littermates ($p = 0.24$). Insets: PPR at inter-stimulus interval of 50 ms in WT littermate and APP/PS1 mice. Hippocampal CA1 neurons from APP/PS1 mice showed similar resting membrane potentials (**C**; $p = 0.47$), rheobases (**D**; $p = 0.78$) and AP amplitudes (**E**; $p = 0.77$) as WT littermate mice. Insets: Single AP firing in CA1 neurons in response to 240-280 pA (for 10 ms) somatic current injection from WT littermate and APP/PS1 animals. Measurement of AP amplitude and after-depolarization. In addition, CA1 cells from adult APP/PS1 mice expressed similar after-depolarizations (**F**; $p = 0.32$), input resistances (**G**; $p = 0.06$), EPSP rise times (**H**; $p = 0.11$) and EPSP decay times (**I**; $p = 0.40$) compared to WT littermate mice. Insets: EPSP time-course, 10%-90% EPSP trace fit for measurement of EPSP rise time and decay time. Digits in the bars represent the number of recorded neurons per condition, at least from three different animals per group. Data expressed as mean ± SEM. Parametric data were compared by two-tailed Student's t-test while non-parametric data were compared by Mann-Whitney U-test.

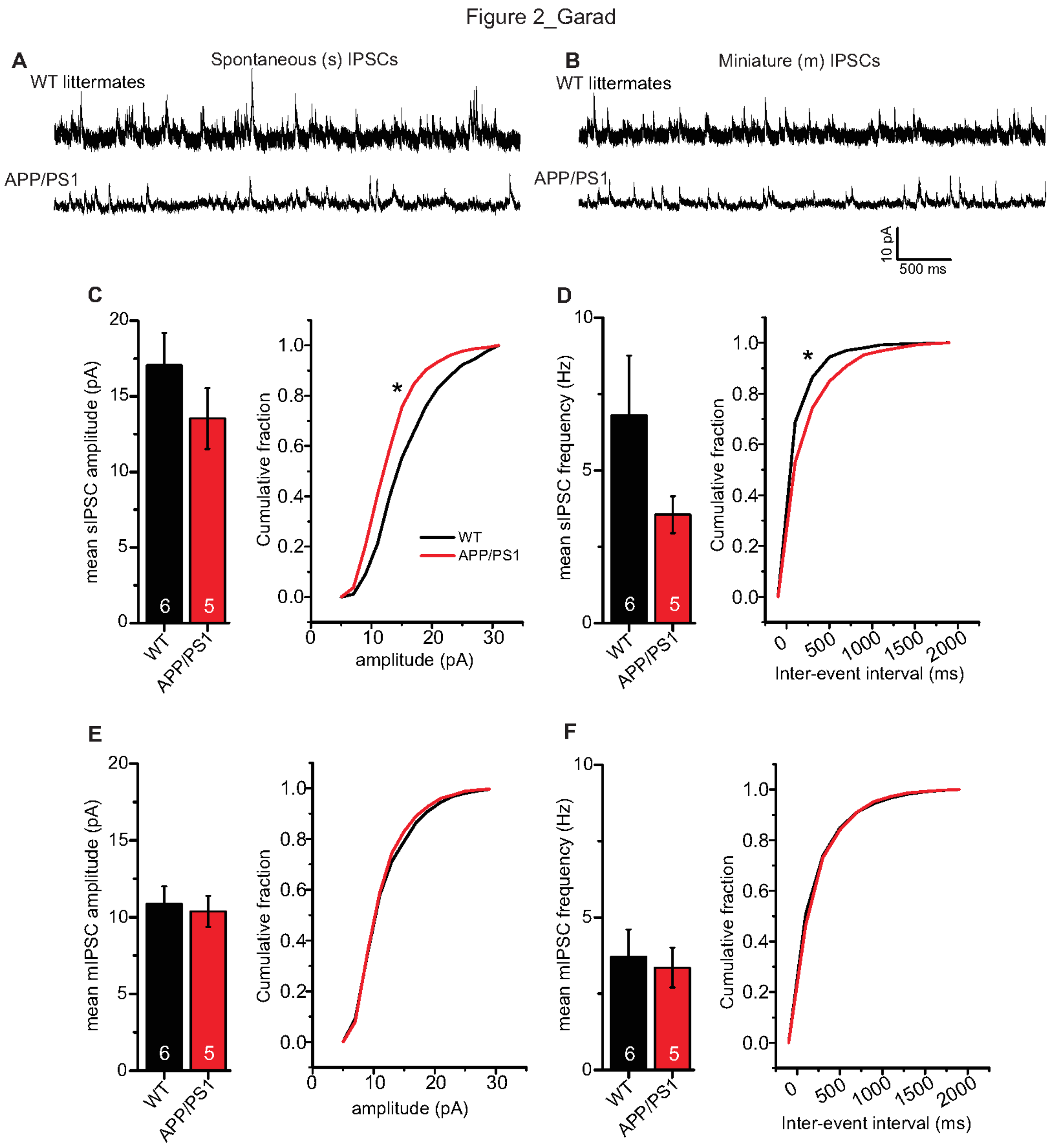

Figure S2: Spontaneous inhibitory synaptic transmission in adult APP/PS1 mice. APP/PS1 mice showed by red symbols, while WT littermate mice designated by black symbols. (**A**) and (**B**) spontaneous (s) and miniature (m) IPSCs in slices from WT littermate and APP/PS1 animals

respectively. (**C**) APP/PS1 and WT littermate mice expressed comparable mean sIPSC amplitudes (p = 0.27), however significantly different cumulative sIPSC amplitudes (p < 0.0001). (**D**) APP/PS1 mice displayed comparable mean sIPSC frequencies as WT littermate mice (p = 0.14). Nonetheless, APP/PS1 and WT littermates expressed significantly different sIPSC inter-event interval (IEI) distribution (p < 0.0001). (**E**) Both WT littermate and APP/PS1 mice showed similar mean mIPSC amplitudes (p = 0.58) and cumulative mIPSC amplitudes (p = 0.13). (**F**) APP/PS1 animals displayed similar mean mIPSC frequencies as WT littermates (p = 0.77), nonetheless significantly different distribution of mIPSC IEI (p = 0.03). Digits in the bars represent the number of recorded neurons per condition, at least from two different animals per group. Data expressed as mean ± SEM. *: p < 0.05, statistics were performed with two-tailed Student's t-test for parametric data and Mann-Whitney U-test for non-parametric data. Cumulative frequency distributions were analyzed with Kolmogorov-Smirnov test.

Supplementary Methods

Whole cell IPSC recordings

Spontaneous and miniature inhibitory postsynaptic currents (IPSCs) were studied in hippocampal CA1 pyramidal neurons in acute slices with no CA3-CA1 cut, prepared from APP/PS1 and WT littermate mice brains as described above. IPSCs were recorded with the intracellular solution containing the following (in mM): 125 Cs-methanosulfonate, 10 CsCl, 2 $MgCl_2$, 5 NaCl, 10 HEPES, 1 EGTA, 5 Mg-ATP and 0.3 Na3GTP; pH was adjusted to 7.2-7.4 using 1 M CsOH [67]. Spontaneous IPSCs were examined in the presence of the N-methyl-d-aspartate receptor (NMDAR) antagonist D,L-2-amino-5-phosphonopentanoic acid (APV, 50 µM, Tocris,

Wiesbaden, Germany) and the α-amino-3-hydroxy-5-methyl-4-isoxazolepropionic acid receptor (AMPAR) antagonist 6,7-dinitroquinoxaline-2,3-dione (DNQX, 10 µM, Tocris, Wiesbaden, Germany) in the extracellular aCSF. Miniature IPSCs were investigated in the presence of APV (50 µM), DNQX (10 µM) and TTX (1 µM) in the extracellular aCSF. IPSCs were recorded at -30 mV holding potential, for the period of 10 min; 250 events from each sweep were used for data analyses. The temperature of external solution in the recording chamber was maintained at 34-36 ^{o}C (aCSF perfusion rate- 2.4 mL/min) for IPSC recordings. Data were analyzed manually using the ‘Mini Analysis program’ (version 6.0.7, Synaptosoft, Decatur, GA, USA).
